# Epstein-Barr Virus Reactivation After Paediatric Haematopoietic Stem Cell Transplantation: Risk Factors and Sensitivity Analysis of Mathematical Model

**DOI:** 10.3389/fimmu.2022.903063

**Published:** 2022-07-12

**Authors:** Soumya P. Kania, Juliana M. F. Silva, Oscar J. Charles, John Booth, S. Y. Amy Cheung, James W. T. Yates, Austen Worth, Judith Breuer, Nigel Klein, Persis J. Amrolia, Paul Veys, Joseph F. Standing

**Affiliations:** ^1^ Infection, Immunity and Inflammation Research & Teaching Department, University College London (UCL) Great Ormond Street Institute of Child Health, University College London, London, United Kingdom; ^2^ Department of Bone Marrow Transplantation, Great Ormond Street Hospital for Children, London, United Kingdom; ^3^ Digital Research, Informatics and Virtual Environment Unit, National Institute for Health and Care Research (NIHR) Great Ormond Street Hospital Biomedical Research Centre, London, United Kingdom; ^4^ Integrated Drug Development, Certara, Princeton, NJ, United States; ^5^ Drug Metabolism and Pharmacokinetics (DMPK) Modelling, In-Vitro In-Vivo Translation, GlaxoSmithKline, Stevenage, United Kingdom; ^6^ Department of Pharmacy, Great Ormond Street Hospital for Children, London, United Kingdom

**Keywords:** viral reactivation, viral kinetics, paediatrics, immune reconstitution, Epstein-Barr virus, haematopoietic stem cell transplant, mathematical modelling

## Abstract

Epstein-Barr virus (EBV) establishes a lifelong latent infection in healthy humans, kept under immune control by cytotoxic T cells (CTLs). Following paediatric haematopoetic stem cell transplantation (HSCT), a loss of immune surveillance leads to opportunistic outgrowth of EBV-infected cells, resulting in EBV reactivation, which can ultimately progress to post-transplant lymphoproliferative disorder (PTLD). The aims of this study were to identify risk factors for EBV reactivation in children in the first 100 days post-HSCT and to assess the suitability of a previously reported mathematical model to mechanistically model EBV reactivation kinetics in this cohort. Retrospective electronic data were collected from 56 children who underwent HSCT at Great Ormond Street Hospital (GOSH) between 2005 and 2016. Using EBV viral load (VL) measurements from weekly quantitative PCR (qPCR) monitoring post-HSCT, a multivariable Cox proportional hazards (Cox-PH) model was developed to assess time to first EBV reactivation event in the first 100 days post-HSCT. Sensitivity analysis of a previously reported mathematical model was performed to identify key parameters affecting EBV VL. Cox-PH modelling revealed EBV seropositivity of the HSCT recipient and administration of anti-thymocyte globulin (ATG) pre-HSCT to be significantly associated with an increased risk of EBV reactivation in the first 100 days post-HSCT (adjusted hazard ratio (AHR) = 2.32, P = 0.02; AHR = 2.55, P = 0.04). Five parameters were found to affect EBV VL in sensitivity analysis of the previously reported mathematical model. In conclusion, we have assessed the effect of multiple covariates on EBV reactivation in the first 100 days post-HSCT in children and have identified key parameters in a previously reported mechanistic mathematical model that affect EBV VL. Future work will aim to fit this model to patient EBV VLs, develop the model to account for interindividual variability and model the effect of clinically relevant covariates such as rituximab therapy and ATG on EBV VL.

## Introduction

Epstein-Barr virus (EBV) is a part of the Herpesviridae family of viruses well-known for their propensity to establish lifelong infections in the human host. To evade the host immune response and establish persistence, EBV expresses latency-associated genes, which allow the virus to reside in resting memory B cells in peripheral blood and maintain a stable long-term viral reservoir (1). In immunocompetent hosts, a circulating pool of EBV-specific cytotoxic T cells (CTLs) is sufficient to control the virus (2). Following allogeneic paediatric haematopoietic stem cell transplantation (HSCT), a loss of immune surveillance results in an opportunistic outgrowth of proliferating EBV-infected B cells leading to EBV reactivation. In the post-HSCT setting, EBV reactivation is the leading cause of post-transplant lymphoproliferative disorder (PTLD) with patients at higher risk if they receive a reduced intensity conditioning (RIC) regimen, selective T cell depletion with anti-thymocyte globulin (ATG) or have low histocompatibility with their donor (3–5). As a part of routine clinical monitoring post-HSCT, quantitative PCR (qPCR) is used to measure EBV DNA and quantify viral load (VL), especially in the first three months when reactivation is most likely to occur. Current treatment protocols for EBV reactivation post-HSCT combine rituximab administration to deplete B cells and reduction of immunosuppression to restore immunity but still counter graft rejection. Studying the kinetics of EBV reactivation in post-HSCT patients at a time when their immune system is reconstituting presents an opportunity to elucidate the biological mechanisms underlying EBV reactivation (6). In this pursuit, mathematical models can be fitted to patients’ EBV VL measurements to estimate biologically meaningful parameters related to the virus, host immune response and effects of therapy to better understand the key drivers of EBV VL (7, 8).

This study aimed to first identify risk factors for EBV reactivation in a paediatric post-HSCT cohort in the first 100 days post-HSCT and secondly, to assess the suitability of a previously reported mathematical model to mechanistically model EBV reactivation kinetics in this population (9).

## Materials and Methods

### Patients and Data Collection

Retrospective electronic data from routine clinical practice were collected from 56 children who underwent HSCT at Great Ormond Street Hospital (GOSH) between 2005 and 2016 and had EBV reactivation post-HSCT. Data were collected by the HSCT clinical team and were subsequently extracted by the GOSH Digital Research Environment (DRE) team. Data collected included patient-specific, donor-specific, and transplant-specific variables as well as measurements of immune cell subsets by immunophenotyping, EBV qPCR and EBV VL, and administrations of alemtuzumab, ATG and rituximab.

### Monitoring EBV Viraemia and Rituximab Therapy

EBV was monitored by qPCR weekly from the start of conditioning until the CD4^+^ T cell count exceeded 300 cells per μl blood. If EBV DNA was detected in the first three months post-HSCT, qPCR was carried out twice weekly to quantify VL until treatment was given or VL declined. The lower and upper limits of quantification of the assay for EBV VL measurements were 200 and 20,000,000 copies/mL respectively. The threshold to treat with rituximab was exceeding EBV VL of 40,000 copies/mL whole blood on two consecutive occasions within the first three months of HSCT, donor different from matched sibling donor (MSD) and a CD3^+^ T cell count < 0.3 x 10^9^/L. Rituximab was dosed by body-surface area and administered *via* intravenous infusion at a dose of 375 mg/m^2^ weekly, with patients receiving a single dose on a conservative regimen or four doses on a pre-emptive regimen. Patients who received four rituximab doses were part of a historical cohort treated using a pre-emptive strategy as part of a study by Worth et al. (10).

### Analysis of Time to First EBV Reactivation Event Using Cox Proportional Hazards Modelling

Cox proportional hazards (Cox-PH) modelling was performed in R version 3.5.1 using the *survival (version 2.42-3)* package to assess time to first EBV reactivation event in the first 100 days post-HSCT (11). The following covariates were considered: type of donor, HSC source, whether the patient had a diagnosis of primary immunodeficiency (PID), age, number of rituximab doses, EBV serostatus of both donor and recipient, type of conditioning regimen, administration of alemtuzumab or ATG as serotherapy pre-HSCT, and area under the curve from 0 to 100 days post-HSCT (AUC_0-100_) for the following immune cell subsets; absolute lymphocyte count (ALC), CD19^+^ B cells, CD4^+^ T cells and CD8^+^ T cells. Collinearity between covariates was assessed before analysis. Variables significant in univariate analysis (P < 0.05) were taken forward to a multivariable analysis.

### Sensitivity Analysis of Mathematical Model of EBV Viral Kinetics

**Figure 1:**
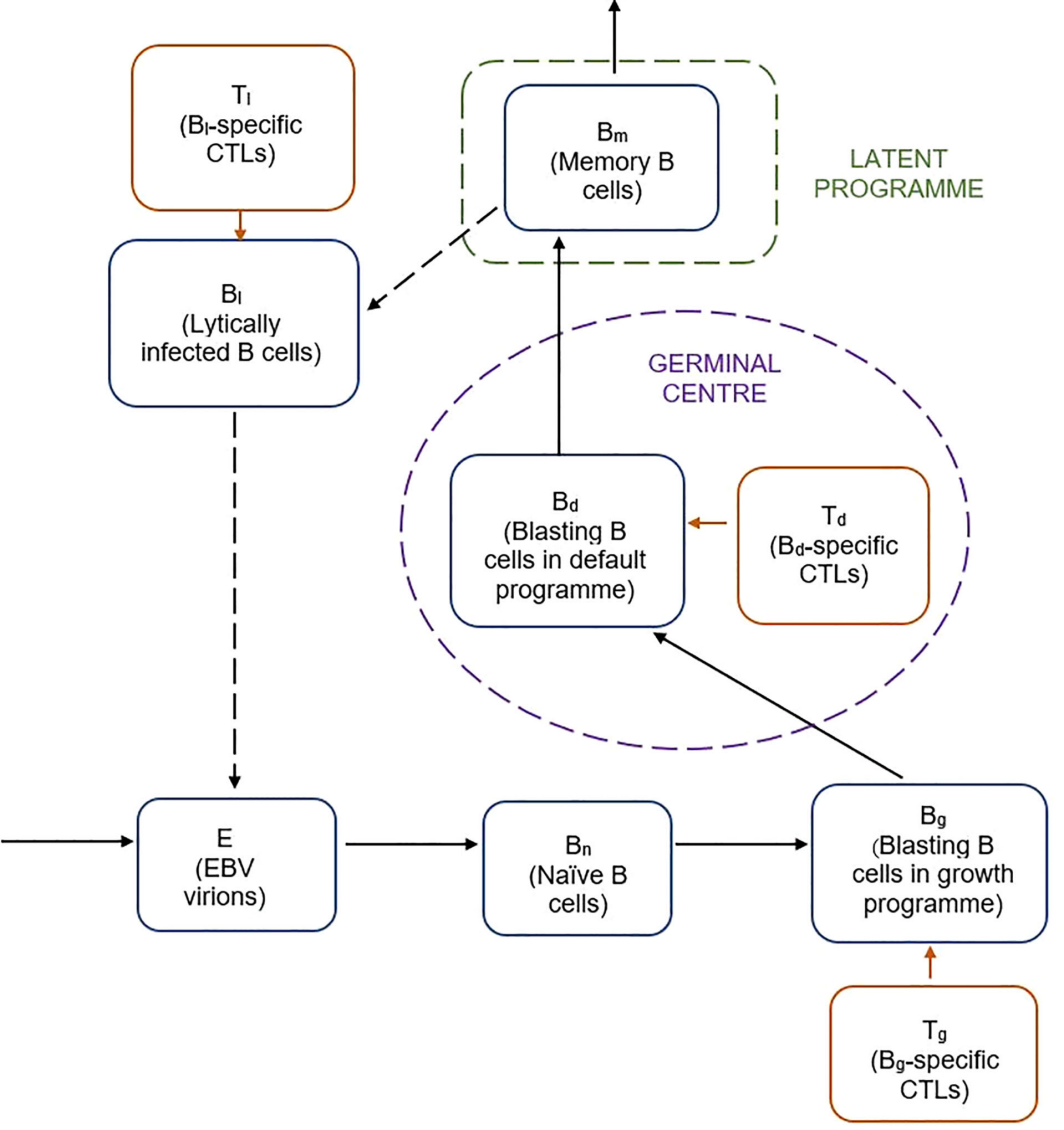
Schematic of previously reported mathematical model of EBV viral kinetics (9).

A previously reported 9-compartment 25-parameter mechanistic mathematical model of EBV viral kinetics was implemented in R version 3.5.1 using the *deSolve* (version 1.28) package (11) (**Figure 1**).

The model describes the key stages of the EBV infection cycle based on the germinal centre theory of EBV biology. It is comprised of nine variables related to the processes of latent and lytic EBV infection, as well as the various types of B cell and CTL involved in the host immune response. Free EBV virions, *E*, infect naive B cells, *B_n_*, of which a fraction transforms into infected B cells, *B_g_*, expressing the growth programme. The remainder proliferate into infected B cells that undergo the default programme in the germinal centre, *B_d_*. Some of the default B cells become latently infected memory B cells, *B_m_*, which can reactivate to become lytically infected B cells, *B_l_*. CTLs specific to the *B_g_, B_d_* and *B_l_* infected B cell populations are included by way of the *T_g_, T_d_* and *T_l_* populations respectively. The system of nine ordinary differential equations for the model are given below:


$$\frac{dE}{dt}=n\delta_{l}B_{l}-\delta_{e}E,$$

$$\frac{dB_{n}}{dt}=\lambda_{n}-\mu_{e}EB_{n}-\delta_{n}B_{n},$$

$$\frac{dB_{g}}{dt}=\left(1-\beta \right)\mu_{e}EB_{n}+\left(r_{g}-\omega_{g}-\delta_{g}\right)B_{g}-\delta_{1}T_{g}B_{g},$$

$$\frac{dB_{d}}{dt}=\omega_{g}B_{g}+\left(r_{d}-\delta_{d}-\omega_{d}\right)B_{d}-\delta_{2}T_{d}B_{d},$$

$$\frac{dB_{m}}{dt}=\omega_{d}B_{d}+\left(r_{m}-\omega_{m}-\delta_{m}\right)B_{m},$$

$$\frac{dB_{l}}{dt}=\omega_{m}B_{m}-\delta_{l}B_{l}-\delta_{3}T_{l}B_{l},$$

$$\frac{dT_{g}}{dt}=r_{1}T_{g}B_{g}-d_{1}T_{g},$$

$$\frac{dT_{d}}{dt}=r_{2}T_{d}B_{d}-d_{2}T_{d},$$

$$\frac{dT_{l}}{dt}=r_{3}T_{l}B_{l}-d_{3}T_{l}.$$

In total, there are 25 parameters, which we fixed to published values to obtain a reference fit of the model (**Supplementary Table 1**) (12–14). The latently infected memory B cell compartment, *B_m_*, was taken as a proxy for EBV VL. The VL sensitivity to each of the 25 parameters was determined by setting each in turn through a range of values (0.00001-1000) to simulate the EBV VL trajectory. The root mean squared distance (RMSD) was calculated for each parameter for the range of parameter values tested to provide a comparison between the reference fit and each of the simulated fits.

## Results

### Patient Characteristics

Data from 56 children were included in this study, with a median age at HSCT of 3.02 years (range, 0.3 - 14 years). The patients were representative of a typical paediatric HSCT cohort, with a range of HSCT indications including malignant and non-malignant conditions, and different types of donor and conditioning regimen. Patient characteristics are summarised in **Table 1**.

**Table 1:** Patient and transplant characteristics.

	Total patients(n = 56)
Age (years) – median (range)	3.0 (0.3 – 14.0)
Diagnosis, n (%)	
PID	19 (33.9)
NMH	12 (21.4)
MH	15 (26.8)
Other	10 (17.9)
Donor type, n (%)	
MSD	6 (10.7)
MFD	4 (7.1)
MUD	28 (50.0)
MMFD	1 (1.8)
MMUD	15 (26.8)
Haplo	2 (3.6)
Stem cell source, n (%)	
BM	32 (57.1)
PBSC	24 (42.9)
Conditioning, n (%)	
MAC	30 (53.6)
MIC	4 (7.1)
RIC	21 (37.5)
None	1 (1.8)
Serotherapy*, n (%)	
Alemtuzumab	40 (72.7)
ATG	15 (27.3)

PID, primary immunodeficiency; NMH, non-malignant haematological; MH, malignant haematological; MSD, matched sibling donor; MFD, matched familial donor; MUD, matched unrelated donor; MMFD, mismatched familial donor; MMUD, mismatched unrelated donor; Haplo, haploidentical donor; BM, bone marrow; PBSC, peripheral blood stem cell; MAC, myeloablative conditioning; MIC, minimal-intensity conditioning; RIC, reduced-intensity conditioning; ATG, anti-thymocyte globulin. *denotes one patient who did not receive serotherapy.

### EBV Reactivation, Clinical Outcomes and Rituximab Therapy

For these 56 patients, 3547 measurements of EBV VL were collected. Of the total patients, 38 (67.9%) had an initial EBV reactivation in the first 100 days since HSCT, with a median time to EBV reactivation of 40 days (range, 14-97 days) and a median peak EBV VL of 255,000 copies/mL (range, 403-32,479,800 copies/mL). Forty-one patients received a single dose of rituximab on a conservative regimen and 15 patients received four doses on a pre-emptive regimen. There were no deaths due to EBV reactivation, or any other reasons, in the study period. Although they did not have an EBV reactivation event in the study period, one patient did progress to PTLD 168 days post-HSCT and started rituximab treatment the following day. This patient had X-linked lymphoproliferative disease, was 7.83 years at HSCT and had the following features predisposing to EBV: received RIC regimen of alemtuzumab, fludarabine and melphalan; the HSC source was PBSC; had a mismatched unrelated donor.

### Visualisations of EBV VL, CD19^+^ B Cells, CD4^+^ Cells and Rituximab Dosing

**Figure 2:**
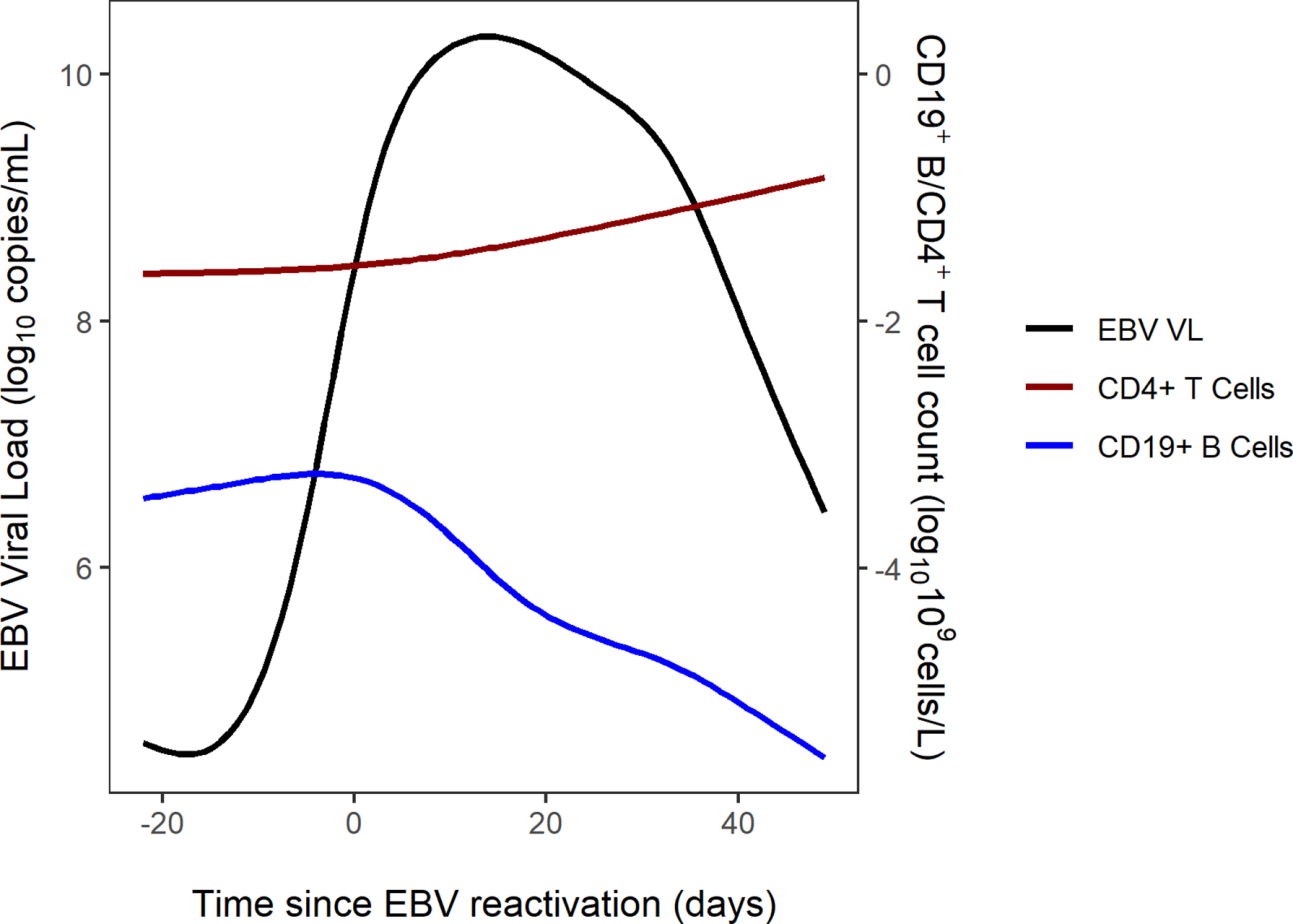
Combined trajectories of EBV VL, CD19^+^ B cell and CD4^+^ T cell counts for 16 out of 56 patients.

**Figure 3:**
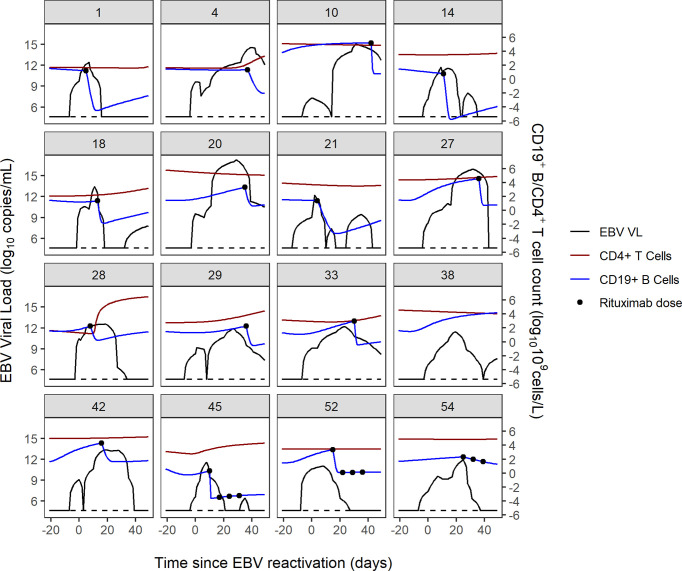
Individual trajectories of EBV VL, CD19^+^ B cell and CD4^+^ T cell counts and rituximab dosing for 16 patients. Dotted black line represents the lower limit of detection of the EBV VL assay (200 copies/mL).

The combined trajectories of EBV VL, CD19^+^ B cells and CD4^+^ cells for 16 of 56 study patients have been visualised in **Figure 2**. Due to patients being lymphopenic in this early post-HSCT period of 100 days, there was a lack of observed CD19^+^ B cell and CD4^+^ T cell counts in our dataset. Therefore, we utilised two previously developed mathematical models to predict CD4^+^ T and CD19^+^ B cells for the first 100 days post-HSCT. CD4^+^ T cell counts were predicted using a published mathematical model for CD4^+^ T cell reconstitution post-HSCT in children and CD19^+^ B cell counts were predicted using a mathematical model for the effect of rituximab on CD19^+^ B cell reconstitution post-HSCT in children (15; unpublished). With respect to EBV VL, observed measurements spanning from three weeks pre- to seven weeks post-HSCT were plotted, and were linearly interpolated to obtain a continuous range of data points for 0-100 days post-HSCT. In the absence of a published mathematical model for CD8^+^ T cell reconstitution post-HSCT and a sparsity of observed CD8^+^ T cell counts disabling linear interpolation, CD8^+^ T cell counts were not visualised. For each of the 16 patients in **Figure 2**, their individual trajectories of EBV VL, CD19^+^ B cells and CD4^+^ T cells as well as rituximab dosing are shown in **Figure 3**. Following administration of the first rituximab dose near the peak EBV VL, CD19^+^ B cell counts rapidly decrease while EBV VL declines simultaneously in all patients. In contrast, CD4^+^ T cell counts remain stable in most patients for the duration of the reactivation event.

### Pre-HSCT ATG and Recipient EBV Seropositivity Increased Risk of EBV Reactivation

**Figure 4:**
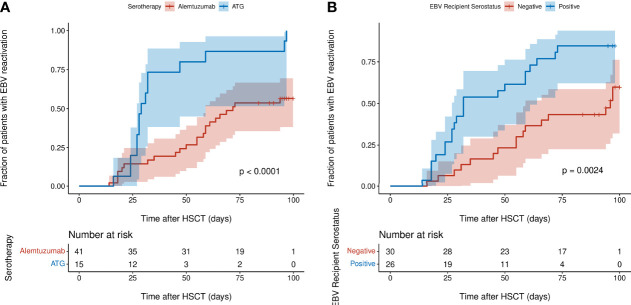
Kaplan-Meier curves of cumulative fraction of patients with EBV reactivation in first 100 days post-HSCT stratified by: **(A)** type of serotherapy (ATG or alemtuzumab) **(B)** EBV serostatus of recipient (negative or positive). *P* values were calculated using the log-rank test and denote the difference between the two subgroups. Shaded regions show 95% confidence interval.

Univariate and multivariable Cox-PH modelling results are shown in **Table 2**. In univariate analysis, a PID diagnosis, AUC_0-100_ of CD8^+^ cells, serotherapy with ATG, HSC source of peripheral blood and EBV seropositivity of HSCT recipient were significantly associated with increasing the risk of EBV reactivation. Of these, two covariates were found to significantly increase risk of EBV reactivation in multivariable analysis; EBV seropositivity of the HSCT recipient and pre-HSCT administration of ATG (adjusted hazard ratio (AHR) = 2.32, *P* = 0.02; AHR = 2.55, *P* = 0.04). Kaplan-Meier curves of the cumulative fraction of patients with EBV reactivation stratified by these two covariates have been visualised in **Figure 4**.

**Table 2:** Univariate and multivariable Cox proportional hazards models for time to first EBV reactivation in first 100 days post-HSCT.

Univariable Model					
Covariates	Term (Reference)	*P* value	HR		95% CI
PID diagnosis	Yes (No)	0.01	0.37		0.17 - 0.82
HSC source	PBSC (BM)	0.02	0.44		0.22 - 0.87
Age	–	0.41	1.03		0.95 - 1.12
Number of rituximab doses	–	0.25	0.86		0.67 - 1.11
Donor EBV serostatus	Seropositive (Seronegative)	0.83	1.17		0.28 - 4.87
Recipient EBV serostatus	Seropositive (Seronegative)	0.003	2.67		1.39 - 5.12
AUC_0-100_ ALC	–	0.90	0.99		0.83 - 1.18
AUC_0-100_ CD19	–	0.65	0.99		0.95 - 1.03
AUC_0-100_ CD4	–	0.77	0.99		0.96 - 1.03
AUC_0-100_ CD8	–	0.005	1.02		1.01 - 1.04
Donor type	MMFD/MMUD/Haplo (MFD/MSD) MUD (MFD/MSD)	0.11 0.27	0.43 1.58		0.16 - 1.21 0.71 - 3.56
Conditioning regimen	RIC (MIC/MAC/None)	0.46	0.78		0.41 - 1.50
Serotherapy	ATG (Alemtuzumab)	0.0001	3.74		1.90 - 7.36
**Multivariable Model**					
**Covariates**	**Term (Reference)**	***P* value**	**HR**		**95% CI**
PID diagnosis	Yes (No)	0.10	0.48		0.20 - 1.14
HSC source	PBSC (BM)	0.48	0.74		0.32 - 1.70
Recipient EBV serostatus	Seropositive (Seronegative)	0.02	2.33		1.15 - 4.73
AUC_0-100_ CD8	–	0.20	1.01		0.99 - 1.03
Serotherapy	ATG (Alemtuzumab)	0.04	2.55		1.07 - 6.11

PID, primary immunodeficiency; HSC, haematopoietic stem cell; PBSC, peripheral blood stem cell; EBV, Epstein-Barr virus; AUC_0-100_, area under the curve from day of HSCT to 100 days post-HSCT; ALC, absolute lymphocyte count; MSD, matched sibling donor; MFD, matched familial donor; MUD, matched unrelated donor; MMFD, mismatched familial donor; MMUD, mismatched unrelated donor; Haplo, haploidentical donor; MAC, myeloablative conditioning; MIC, minimal-intensity conditioning; RIC, reduced-intensity conditioning; ATG, anti-thymocyte globulin. HR, hazard ratio; CI, confidence interval. – denotes a continuous variable.

### Parameters Related to Latently Infected Memory B Cells and CTLs Important for EBV VL

**Figure 5:**
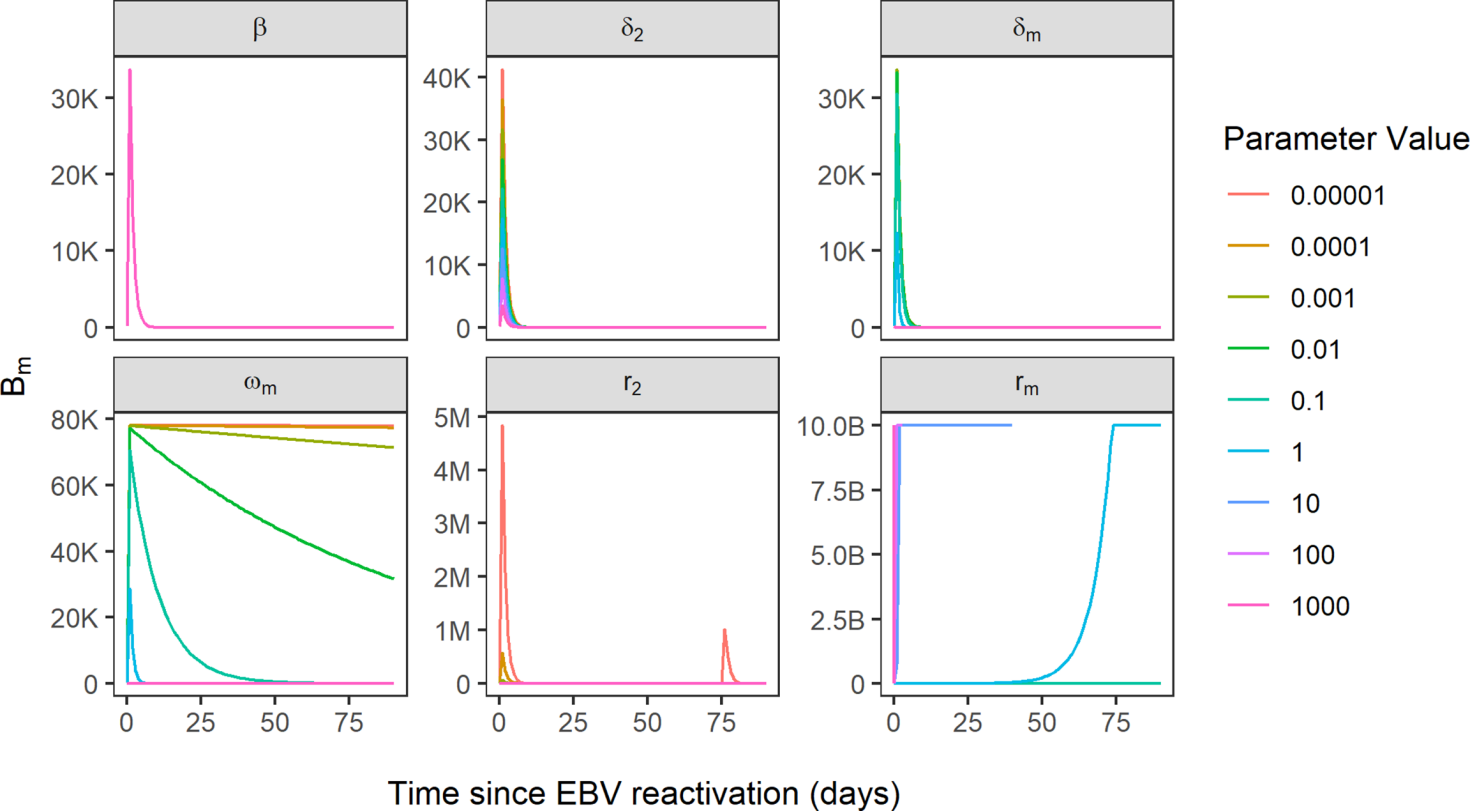
Simulated EBV viral load trajectories, using B_m_ compartment as a proxy for EBV VL. Sensitivity of EBV VL shown for the variation of six parameters over the range 0.00001-1000, where beta parameter represents reference model fit and all other parameters are sensitive parameters. B, 1 billion; M, 1 million; K, 1000.

Of the 25 parameters in the model, 13 parameters were found to be sensitive on account of an RMSD value greater than zero (**Supplementary Table 2**). Coupled with visual assessment of the B_m_ compartment plotted by time for each of the parameters, the following five of the 13 parameters in the model were identified as being key determinants of EBV VL: δ_2_, CTL killing rate of infected B cells expressing the default programme; r_m_, the proliferation rate of latently infected memory B cells; ω_m_, the reactivation rate of latently infected memory B cells into lytically infected memory B cells; d_m_, the death rate of latently infected memory B cells and r_2_, the rate of CTL activation against infected B cells expressing the default programme (**Figure 5**). Of these five parameters, three are related to latently infected memory B cells and two are related to CTLs.

## Discussion

In this retrospective study, we tested the effect of multiple predictor variables for EBV reactivation in a paediatric cohort of post-HSCT patients. We found pre-HSCT ATG administration and EBV seropositivity of the HSCT recipient to be significant risk factors for the first EBV reactivation event in the 100-day period post-HSCT. In addition, for the first time, we have identified key parameters driving EBV VL in the previously reported mechanistic mathematical model of EBV viral kinetics by Akinwumi, which may have potential to describe EBV VL data from patients with EBV reactivation post-HSCT.

Pre-HSCT ATG has been widely reported to be associated with higher incidence of EBV reactivation post-HSCT therefore our result corroborates these previous findings (5, 16–18). Even though it is administered as part of the conditioning regimen for GvHD prophylaxis in the weeks pre-HSCT, ATG’s T-cell depletive effect lasts well into the early post-HSCT period due to its long half-life of 29.8 days (16). This may further exacerbate the reduced CTL-mediated immune surveillance of EBV post-HSCT when the CD8^+^ T cell compartment is reconstituting. In our study, 100% (15 of 15) of patients who received ATG had EBV reactivation in the first 100 days post-HSCT while only 57.5% (23 of 40) of patients who received alemtuzumab had EBV reactivation, a finding comparable with previous studies, most probably attributed to alemtuzumab’s broader lymphocyte depletion of B cells, including those that are EBV-infected (3, 5, 19, 20).

Although the historical consensus is that EBV serological donor/recipient mismatch increases risk of EBV reactivation, most studies ascribe EBV-associated PTLD to donor-derived EBV, which contradicts our finding that EBV seropositivity of the HSCT recipient is a risk factor for EBV reactivation (21, 22). As the focus of our study was on the first EBV reactivation event post-HSCT, we chose a study period of the first 100 days post-HSCT when reactivation is most likely to occur. In contrast, many studies of EBV reactivation have much longer study periods spanning from six months to nearly five years post-HSCT (10, 23–26). At these later timepoints post-HSCT, there would be more donor-derived cells due to increased chimerism, which we might not have been able to capture in our study period of 100 days post-HSCT. This is also evident when looking at the EBV serological donor/recipient combinations in our patients; in EBV seropositive donors with EBV seronegative recipients, 55.2% (16 of 29) of patients had an EBV reactivation while 87.0% (20 of 23) of patients who were EBV-seropositive and had EBV-seropositive donors experienced an EBV reactivation event. There were only three patients who were EBV seropositive and had EBV seronegative donors, of which two had EBV reactivation. Having access to chimerism data at post-HSCT timepoints for our study patients might help to shed further light on this result.

The incidence of EBV reactivation in our study of 67.9% is high but similar to that observed in some previous studies (27, 28). This may be expected as the first 100 days post-HSCT is when EBV reactivation is most likely to occur due to insufficient T cell reconstitution of the patient’s immune system to control EBV. In addition, it is recognised that the incidence of EBV reactivation post-HSCT varies depending on the transplant type, sensitivity of the EBV quantification assay, definitions of thresholds of EBV viraemia and timing of reactivation (29).

EBV reactivation in patients post-HSCT occurs in the same timeframe that patients’ immune systems are reconstituting presenting an opportunity to elucidate the biological mechanisms underlying EBV reactivation in this setting. The visualised trajectories of EBV VL, CD19^+^ B cells and CD4^+^ T cells in response to EBV and rituximab for individual patients give an insight into the pattern of EBV reactivation for each patient, demonstrating the complexity in the dynamics of post-HSCT EBV reactivation and its variability between patients. The decrease in B cell count occurs shortly after the first rituximab dose is administered, as per the B-cell depleting mechanism of action of rituximab and is coupled with a contemporaneous decrease in EBV VL in all patients. Conversely, CD4^+^ T cell counts remain stable in most patients during the reactivation, which aligns with a previous study which reported no increase in CD4^+^ T cell counts after viral reactivation (23). In addition, we can infer from **Figure 3** that subsequent rituximab doses in patients may not further decrease B cell count and consequently EBV VL, as suggested by patients 45, 52 and 54. In producing these figures, we demonstrate the ability of mathematical models to predict immune reconstitution after insult to the immune system, such as viral reactivation post-HSCT. Mathematical modelling is a suitable approach to study this, as it can capture the non-linearity of the underlying biological processes and the heterogeneity observed in EBV reactivation due to the influence of patient-, donor- transplant-, disease- and drug-related factors (30). Our main finding from performing sensitivity analysis of the model of Akinwumi, that parameters related to latent memory B cells and CTLs determine changes in EBV VL, is aligned with the key role that these two cell types have in the biological mechanism of EBV reactivation. This corroborates the usefulness of a mathematical modelling approach to further quantify the kinetics of EBV reactivation in patients. Further, this finding aligns with a study by Burns et al., who observed an increase in number and proportion of CD27^+^ memory B cells in peripheral blood samples of post-HSCT patients that were preferentially infected by EBV in a latent form and expressed the cell proliferation marker Ki-67 (6).

We acknowledge the limitations of our work. The small sample size of 56 patients is characteristic of the study being retrospective and based at a single centre as well as the outcome of interest, EBV reactivation in children post-HSCT, being uncommon. Nevertheless, other authors have also conducted multivariable analysis on data from a comparable number or fewer patients in the context of EBV reactivation (19, 31, 32). Another limitation is the lack of observed immune cell counts for the early post-HSCT period studied, which we were partly able to circumvent by leveraging previously developed mathematical models by our group. In addition, while our data did represent a typical HSCT cohort, there were no patients who underwent HSCT using cord blood. The pattern of EBV reactivation for such patients may differ considerably to patients transplanted using PBSCs or BM, as their T cell reconstitution has been reported to be much faster, attributed to cord blood HSCT patients receiving ATG-free pre-HSCT conditioning regimens and being of younger age at HSCT (15, 33, 34). The prognostic value of the mathematical model of Akinwumi, or indeed any other mathematical model of EBV kinetics, can only be determined after it is fit to clinical data from patients with EBV reactivation. Therefore, future work will aim to fit the previously reported model to the observed EBV VL measurements of the paediatric patients in this study cohort. With respect to the mathematical model of Akinwumi, it may be unfeasible to fit such a complex model to estimate parameters from patient EBV VLs therefore a simplified version of the model may be more appropriate. As we move forward, it will be important to include the covariates from the Cox-PH model into the mechanistic model-building process. At this stage prior biological knowledge related to B cell maturation can be used to scale for age-related effects, helping to account for the variability seen in the EBV VL trajectories of individual patients.

In conclusion, we have consolidated previous findings by assessing the effect of multiple risk factors on EBV reactivation in the first 100 days post-HSCT in children. In addition, we demonstrate the applicability of a mathematical modelling approach to describe patient’s EBV VL trajectories by identifying key determinants of EBV VL in a previously reported mechanistic mathematical model. Mechanistically modelling patient EBV VL data will allow us to delineate and quantify the viral, drug and immune mechanisms at play in this cohort of patients. Ultimately, this would inform the clinical management of this cohort of patients, as a mathematical model can be used to make inferences on treatment, such as the timing of rituximab therapy, to improve outcomes of patients with EBV reactivation post-HSCT.

## Data Availability Statement

The raw data supporting the conclusions of this article will be made available by the authors, without undue reservation.

## Ethics Statement

This study involved human participants and was issued ethical approval by the Great Ormond Street Hospital for Children (GOSH) NHS Trust for the project “Extrapolation of haematopoiesis dynamics following cytotoxic insult to personalise paediatric drug development” (REC reference 17/LO/0008: Use of routine GOSH data for research, R&D reference 18IR20). In addition, this study utilised the GOSH Digital Research Environment (DRE) with access to previously collected, non-identifiable clinical information. This is covered under the ethical approval 17/LO/0008. Written informed consent from the participants’ legal guardian/next of kin was not required to participate in this study in accordance with the national legislation and the institutional requirements.

## Author Contributions

SPK, SYAC, JWTY and JFS designed the study. JMFS, AW, PJA and PV collected the data. JBo extracted the data. SPK, JMFS and OJC analysed the data. SPK, JMFS, OJC, SYAC, JWTY, JBr, NK and JFS interpreted the data and wrote the manuscript. All authors contributed to the article and approved the submitted version.

## Funding

Support at the institution level came from the National Institute for Health and Care Research Biomedical Research Centre at Great Ormond Street Hospital for Children NHS Foundation Trust and University College London. SPK was funded by a United Kingdom (UK) Medical Research Council (MRC) industrial Collaborative Award in Science and Engineering (iCASE) PhD Studentship (MR/R015759/1) and AstraZeneca. OJC was funded by a UK MRC PhD Studentship (MR/N013867/1).

## Conflict of Interest

JY is employed by GSK.

The remaining authors declare that the research was conducted in the absence of any commercial or financial relationships that could be construed as a potential conflict of interest.

This study received funding from AstraZeneca. The funder was not involved in the study design, collection, analysis, interpretation of data, the writing of this article or the decision to submit it for publication.

## Publisher’s Note

All claims expressed in this article are solely those of the authors and do not necessarily represent those of their affiliated organizations, or those of the publisher, the editors and the reviewers. Any product that may be evaluated in this article, or claim that may be made by its manufacturer, is not guaranteed or endorsed by the publisher.
