# Supplementary material for: Epstein-Barr Virus Reactivation After Paediatric Haematopoietic Stem Cell Transplantation: Risk Factors and Sensitivity Analysis of Mathematical Model
Source: Front Immunol. 2022 Jul 12;13:903063. doi: 10.3389/fimmu.2022.903063 (PMC9314642; doi:10.3389/fimmu.2022.903063)
Supplement: Supplementary file 1 [file DataSheet_1.docx]

**SUPPLEMENTARY MATERIAL:**

**Epstein-Barr Virus Reactivation After Paediatric Haematopoietic Stem Cell Transplantation: Risk Factors and Sensitivity Analysis of Mathematical Model**

**Supplementary Table 1:** Parameter values of mathematical model of EBV viral kinetics

| **Parameter** | **Description** | **Value** | **Reference** |
| --- | --- | --- | --- |
| $n$ | Viral burst size | 1.00 × 10^5^ | 1, 2 |
| $\delta_{l}$ | Death rate of lytically infected cell due to viral burst | 2.32 × 10^−4^ | 1, 2 |
| $\delta_{e}$ | Death rate of EBV | 2.33 | 1 |
| $\lambda_{n}$ | Production rate of naïve B cells | 5.04 × 10^5^ | 1, 2 |
| *β* | NK cell effect on EBV infection | 0.80 | ^a^ |
| $\mu_{e}$ | EBV infection rate per B cell virus | 3.3 × 10^−8^ | 2 |
| $\delta_{n}$ | Death rate of naïve B cells | 1.68 | 2 |
| $r_{g}$ | Proliferation rate of infected B cells expressing growth programme | 37.8 | 1, 2 |
| $\omega_{g}$ | Transit rate from growth compartment to default compartment | 4.90 | 2 |
| $\delta_{g}$ | Death rate of infected B cells expressing the growth programme | 0.88 | 1, 2 |
| $\delta_{1}$ | Rate at which CTL kills infected B cells expressing the growth programme | 3.83 × 10^−4^ | 2 |
| $r_{d}$ | Proliferation rate of infected B cells expressing the default programme | 38.81 | 3 |
| $\omega_{d}$ | Transit rate from default compartment to memory compartment | 10.08 | 2, 3 |
| $\delta_{d}$ | Death rate of infected germinal B cells expressing the default program | 10.08 | 2, 3 |
| $\delta_{2}$ | Rate at which CTL kills infected B cells expressing the default program | 3.83 × 10^−4^ | 2 |
| $r_{m}$ | Proliferation rate of infected memory B cells | 0.00 | 1, 2 |
| $\omega_{m}$ | Reactivation rate of infected memory B cells into lytically infected B cells | 0.84 | 1, 2 |
| $\delta_{m}$ | Death rate of infected memory B cells | 0.00 | 1, 2, 3 |
| $\delta_{3}$ | Rate at which CTL kills lytically infected B cells | 7.66 × 10^−4^ | 1, 2 |
| $r_{1}$ | Rate of CTL activation against infected B cells expressing the growth program | 1.40 × 10^−3^ | 1, 2 |
| $r_{2}$ | Rate of CTL activation against infected B cells expressing the default programme | 2.10 × 10^−3^ | 1, 2, 4 |
| $r_{3}$ | Rate of CTL activation against lytically infected B cells | 4.90 × 10^−3^ | 1, 2 |
| $d_{1}$ | Death rate of CTLs responding to the growth compartment | 6.46 × 10^−2^ | 1, 4 |
| $d_{2}$ | Death rate of CTLs responding to the default compartment | 6.46 × 10^−2^ | 1, 4 |
| $d_{3}$ | Death rate of CTLs responding to the lytic compartment | 6.46 × 10^−2^ | 1, 4 |

^a^ Assumption; Parameters are measured per week except for *n* and *β* that are dimensionless.

**Supplementary Table 2**: RMSD values from sensitivity analysis of mathematical model of EBV viral kinetics

| **Parameter Name** | **Parameter Value** | **RMSD** |
| --- | --- | --- |
| $\mu_{e}$ | 0.00001 | 0.0000598 |
| $\mu_{e}$ | 0.0001 | 0.0002277 |
| $\mu_{e}$ | 0.001 | 0.0002995 |
| $\mu_{e}$ | 0.01 | 0.0002638 |
| $\mu_{e}$ | 0.1 | 0.0002838 |
| $\mu_{e}$ | 1 | 0.3540263 |
| $\mu_{e}$ | 10 | 664.516682 |
| $\mu_{e}$ | 100 | 664.511591 |
| $\mu_{e}$ | 1000 | 0.4624968 |
| $\omega_{g}$ | 0.00001 | 0.0221101 |
| $\omega_{g}$ | 0.0001 | 0.0220577 |
| $\omega_{g}$ | 0.001 | 0.0220684 |
| $\omega_{g}$ | 0.01 | 0.022036 |
| $\omega_{g}$ | 0.1 | 0.0216765 |
| $\omega_{g}$ | 1 | 0.0175886 |
| $\omega_{g}$ | 10 | 0.0229978 |
| $\omega_{g}$ | 100 | 0.4268789 |
| $\omega_{g}$ | 1000 | 4.2287738 |
| $r_{d}$ | 0.00001 | 0.11996 |
| $r_{d}$ | 0.0001 | 0.1199354 |
| $r_{d}$ | 0.001 | 0.1199499 |
| $r_{d}$ | 0.01 | 0.1199338 |
| $r_{d}$ | 0.1 | 0.119622 |
| $r_{d}$ | 1 | 0.1168459 |
| $r_{d}$ | 10 | 0.0890299 |
| $r_{d}$ | 100 | 0.1886247 |
| $r_{d}$ | 1000 | 2.8748616 |
| $\delta_{d}$ | 0.00001 | 0.0311306 |
| $\delta_{d}$ | 0.0001 | 0.031044 |
| $\delta_{d}$ | 0.001 | 0.0311344 |
| $\delta_{d}$ | 0.01 | 0.0310927 |
| $\delta_{d}$ | 0.1 | 0.0308214 |
| $\delta_{d}$ | 1 | 0.0280641 |
| $\delta_{d}$ | 10 | 0.0002721 |
| $\delta_{d}$ | 100 | 0.2784072 |
| $\delta_{d}$ | 1000 | 3.1629684 |
| $\omega_{d}$ | 0.00001 | 649.653009 |
| $\omega_{d}$ | 0.0001 | 649.647208 |
| $\omega_{d}$ | 0.001 | 649.589201 |
| $\omega_{d}$ | 0.01 | 649.009125 |
| $\omega_{d}$ | 0.1 | 643.208371 |
| $\omega_{d}$ | 1 | 585.201102 |
| $\omega_{d}$ | 10 | 5.1557006 |
| $\omega_{d}$ | 100 | 5792.56153 |
| $\omega_{d}$ | 1000 | 63486.3354 |
| $\delta_{2}$ | 0.00001 | 146.097791 |
| $\delta_{2}$ | 0.0001 | 53.8174597 |
| $\delta_{2}$ | 0.001 | 38.4620936 |
| $\delta_{2}$ | 0.01 | 130.740739 |
| $\delta_{2}$ | 0.1 | 223.01765 |
| $\delta_{2}$ | 1 | 315.286014 |
| $\delta_{2}$ | 10 | 407.476587 |
| $\delta_{2}$ | 100 | 498.904832 |
| $\delta_{2}$ | 1000 | 583.568495 |
| $r_{m}$ | 0.00001 | 0.0115386 |
| $r_{m}$ | 0.0001 | 0.1146481 |
| $r_{m}$ | 0.001 | 1.1476092 |
| $r_{m}$ | 0.01 | 11.607003 |
| $r_{m}$ | 0.1 | 131.001159 |
| $r_{m}$ | 1 | 2553452116 |
| $r_{m}$ | 10 | NA |
| $r_{m}$ | 100 | NA |
| $r_{m}$ | 1000 | 4945054770 |
| $\omega_{m}$ | 0.00001 | 76475.2946 |
| $\omega_{m}$ | 0.0001 | 76160.3638 |
| $\omega_{m}$ | 0.001 | 73103.0948 |
| $\omega_{m}$ | 0.01 | 49972.6235 |
| $\omega_{m}$ | 0.1 | 7499.69306 |
| $\omega_{m}$ | 1 | 152.344669 |
| $\omega_{m}$ | 10 | 651.28431 |
| $\omega_{m}$ | 100 | 651.323251 |
| $\omega_{m}$ | 1000 | 651.323251 |
| $\delta_{m}$ | 0.00001 | 0.0113832 |
| $\delta_{m}$ | 0.0001 | 0.114596 |
| $\delta_{m}$ | 0.001 | 1.144613 |
| $\delta_{m}$ | 0.01 | 11.3181301 |
| $\delta_{m}$ | 0.1 | 101.711695 |
| $\delta_{m}$ | 1 | 489.441266 |
| $\delta_{m}$ | 10 | 651.306439 |
| $\delta_{m}$ | 100 | 651.323251 |
| $\delta_{m}$ | 1000 | 651.323251 |
| $r_{1}$ | 0.00001 | 679.739473 |
| $r_{1}$ | 0.0001 | 0.2361807 |
| $r_{1}$ | 0.001 | 0.0082162 |
| $r_{1}$ | 0.01 | 0.018522 |
| $r_{1}$ | 0.1 | 0.0214479 |
| $r_{1}$ | 1 | 0.0230053 |
| $r_{1}$ | 10 | 0.0230268 |
| $r_{1}$ | 100 | 0.0229884 |
| $r_{1}$ | 1000 | 0.0225305 |
| $d_{1}$ | 0.00001 | 0.0000591 |
| $d_{1}$ | 0.0001 | 0.0000725 |
| $d_{1}$ | 0.001 | 0.0001126 |
| $d_{1}$ | 0.01 | 0.000069 |
| $d_{1}$ | 0.1 | 0.5352592 |
| $d_{1}$ | 1 | 10.3410168 |
| $d_{1}$ | 10 | 63.1074901 |
| $d_{1}$ | 100 | 182.454391 |
| $d_{1}$ | 1000 | 27506.9519 |
| $r_{2}$ | 0.00001 | 113364.74 |
| $r_{2}$ | 0.0001 | 10456.245 |
| $r_{2}$ | 0.001 | 652.349789 |
| $r_{2}$ | 0.01 | 500.106597 |
| $r_{2}$ | 0.1 | 632.761725 |
| $r_{2}$ | 1 | 647.770708 |
| $r_{2}$ | 10 | 649.445983 |
| $r_{2}$ | 100 | 649.630949 |
| $r_{2}$ | 1000 | 649.651189 |
| $d_{2}$ | 0.00001 | 0.0002103 |
| $d_{2}$ | 0.0001 | 0.0002241 |
| $d_{2}$ | 0.001 | 0.0002069 |
| $d_{2}$ | 0.01 | 0.0001969 |
| $d_{2}$ | 0.1 | 0.000189 |
| $d_{2}$ | 1 | 4790.4955 |
| $d_{2}$ | 10 | 56796.5727 |
| $d_{2}$ | 100 | 765878.122 |
| $d_{2}$ | 1000 | 8901197171 |

**References**

1. Shapiro M, Duca KA, Lee K, Delgado-Eckert E, Hawkins J, Jarrah AS, et al. A Virtual Look at Epstein–Barr Virus Infection: Simulation Mechanism. J Theor Biol (2008) 252(4):633–48. doi: 10.1016/j.jtbi.2008.01.032
2. Huynh GT, Adler FR. Alternating host cell tropism shapes the persistence, evolution and coexistence of Epstein–Barr virus infections in human. Bulletin of mathematical biology. 2011 Aug;73(8):1754-73. doi: 10.1007/s11538-010-9590-8
3. Roughan JE, Torgbor C, Thorley-Lawson DA. Germinal Center B Cells Latently Infected With Epstein-Barr Virus Proliferate Extensively But do Not Increase in Number. J Virol (2010) 84(2):1158–68. doi: 10.1128/JVI.01780-09
4. Hawkins JB, Delgado-Eckert E, Thorley-Lawson DA, Shapiro M. The Cycle of EBV Infection Explains Persistence, the Sizes of the Infected Cell Populations and Which Come Under CTL Regulation. PloS Pathog (2013) 9(10):e1003685. doi: 10.1371/journal.ppat.1003685
